# Venovenous Extracorporeal Membrane Oxygenation Therapy in Adults

**DOI:** 10.7759/cureus.5365

**Published:** 2019-08-11

**Authors:** Avani R Patel, Amar R Patel, Shivank Singh, Shantanu Singh, Nancy J Munn

**Affiliations:** 1 Internal Medicine, Northern California Kaiser Permanente, Fremont, USA; 2 Internal Medicine, Southern Medical University, Guangzhou, CHN; 3 Pulmonary Medicine, Marshall University School of Medicine, Huntington, USA; 4 Pulmonary Medicine, Veterans Affairs Medical Center, Huntington, USA

**Keywords:** venovenous extracorporeal membrane oxygenation (vv-ecmo), extracorporeal membrane oxygenation (ecmo), membrane lung, barotrauma, acute respiratory distress syndrome (ards), seldinger technique, semi-seldinger technique, murray score, age-adjusted oxygenation index, apss

## Abstract

Extracorporeal membrane oxygenation (ECMO) therapy is used as supportive therapy for patients with respiratory failure, cardiac failure, and cardiopulmonary failure. Venovenous extracorporeal membrane oxygenation (VV-ECMO) is one subtype used for respiratory failure as a supportive treatment for critically ill patients. The principle behind it is that the membrane lung (oxygenator) is placed sequentially with the normal lungs rather than in parallel like with cardiopulmonary bypass, therefore, the lungs do not have to work as hard to oxygenate the blood. Then using a drainage cannula, blood is drained from the right atrium (RA) and after going through the membrane lung, the newly oxygenated blood is returned back to the RA. Because of this, there is enough systemic oxygen delivery to manage metabolism and preserve the airway even at lower tidal volume ventilation settings. With ventilator settings placed at lower tidal volume, there is less risk of barotrauma. This is a review article discussing VV-ECMO therapy with adult patients. It will also go into detail regarding its indications, contraindications, configurations, patient assessment, vascular access, and complications.

## Introduction and background

Extracorporeal membrane oxygenation (ECMO) therapy has greatly influenced the way physicians treat and manage intensive care unit (ICU) patients. In principle, ECMO therapy is a mechanical circuit that functions outside of the body (extracorporeal) to provide respiratory, cardiac, or cardiopulmonary support. Since the 1970s, ECMO has functioned as supportive therapy for critically ill patients who require functional support but have pathophysiology that is incompletely managed by invasive mechanical ventilation, inotropes, and vasopressors. ECMO therapy equipment consists of a blood pump, a membrane lung (also called an oxygenator), conduit tubing, a heat exchanger, and two cannulas for drainage and blood return. ECMO therapy works by draining blood through a drainage cannula (with blood pump) and then removing the carbon dioxide and adding oxygen to the blood using a membrane lung. The newly oxygenated blood is then returned to the body by a reinfusion cannula. A membrane lung is a gas exchange device that uses a semipermeable membrane to add and remove gases from the blood. By utilizing this oxygenation circuit as a supportive therapy, physicians can provide more effective treatment for patients with respiratory, cardiac, or cardiopulmonary failure. This is because many of those patients are also on ventilators, which need to remain at low tidal volume and lower positive end-expiratory pressure settings in order to prevent further destruction of already damaged lung tissue. This is why ECMO therapy use has drastically increased. One of the subtypes of ECMO therapy is venovenous extracorporeal membrane oxygenation (VV-ECMO) [1]. This is a review article discussing VV-ECMO therapy with adult patients. It will also go into detail regarding VV-ECMO and its indications, contraindications, configurations, patient assessment, vascular access, and complications. Our aim is to spread further awareness regarding VV-ECMO and how it can be of further benefit to patients.

## Review

Principle of venovenous extracorporeal membrane oxygenation therapy

The principle of it is that the membrane lung (oxygenator) is placed sequentially with the normal lungs rather than in parallel like with cardiopulmonary bypass, therefore, the lungs do not have to work as hard to oxygenate the blood [2]. Using a drainage cannula, blood is drained from the right atrium (RA) and after going through the membrane lung, the newly oxygenated blood is returned back to the RA [3]. Because of this, the newly oxygenated blood mixes with the native venous blood and helps provide enough systemic oxygen delivery to manage metabolism and preserve the airway even with at rest ventilation settings. With ventilator settings placed at lower tidal volume, there is less risk of barotrauma.

Indications

The main indication for VV-ECMO therapy is severe respiratory failure that is incompletely treated by optimal mechanical ventilation and medication. In hypoxic respiratory failure, VV-ECMO therapy is indicated when the risk of mortality is greater than or equal to 80% [4]. 

A mortality risk of 80% occurs when a patient develops certain examination findings despite having received optimal care for less than six hours. These findings include a value less than 100 of the partial pressure of oxygen in arterial blood (PaO_2_) divided by the fractional concentration of O_2 _in inspired gas (FiO_2_), the FiO_2 _> 90%, with the patient possibly having a Murray Score 3-4 or the Age-Adjusted Oxygenation Index (AOI) > 80 or the age, PaO_2_/FiO_2 _ratio, and the plateau pressure (APSS) = 8 [4-7].

The Murray Score is used to grade the severity of lung injury in acute respiratory distress syndrome (ARDS). A score greater than 2.5 will indicate ARDS, a score between 1-2.5 indicates mild to moderate lung injury, and a score of zero rules out lung injury [5]. The Murray Score is based on four criteria, which are hypoxemia, respiratory compliance, chest radiographic findings, and the level of positive end-expiratory pressure [5]. Each criterion receives a score from zero to four according to condition severity and those numbers are summed to create the Murray Score [5]. 

The AOI or Age-Adjusted Oxygenation Index is another score used to grade the severity and prognosis of ARDS [6]. The AOI is calculated by measuring the mean airway pressure and multiplying it with the FiO_2_, afterwards the value is divided by the PaO_2_ [6].

The APSS scoring system was created in 2016. It is based on a nine-point grading system that is calculated from the patient’s age, PaO_2_/FiO_2 _ratio, and the plateau pressure 24 hours after ARDS diagnosis [7]. Patients with a score of seven have mortality greater than 80% while patients with scores less than five would have a mortality of about 14% [7].

Other indications include acute respiratory distress syndrome, hypercapnic respiratory failure, severe air leak syndromes, bridge to lung transplantation, and post-transplantation primary graft dysfunction [4].

Contraindications

There are no clear contraindications for VV-ECMO, although certain patient conditions have been categorized as relative contraindications. These conditions are associated with poor outcomes despite the use of VV-ECMO [4]. Relative contraindications include mechanical ventilation at high settings, immunosuppression, recent or expanding CNS hemorrhage, terminal malignancy, and increasing age Table 1.

**Table 1:** The Relative Contraindications for VV-ECMO These are conditions that would be associated with poor outcomes in patients regardless of the use of VV-ECMO [4]. VV-ECMO: venovenous extracorporeal membrane oxygenation; FiO_2_: fractional concentration of O_2 _in inspired gas; mm^3^: millimeters cubed.

Relative Contraindications to VV-ECMO Therapy [4]
Mechanical ventilation at high settings for seven days or more (FiO_2_> 0.9)
Immunosuppression (absolute neutrophil count <400/mm^3^)
Recent or expanding CNS hemorrhage
Major CNS damage or terminal cancer
Increasing age

Configurations of venovenous extracorporeal membrane oxygenation therapy

There are four different configurations of VV-ECMO therapy. They are the femoral-jugular VV-ECMO, the femoral-femoral VV-ECMO, dual lumen cannula VV-ECMO, femoral-femoral-jugular vein-artery-vein extracorporeal membrane oxygenation (VAV-ECMO).

The femoral-jugular VV-ECMO is known as the classic configuration of VV-ECMO. The drainage cannula (inflow) is inserted into the femoral vein and advanced upwards till the cannula tip reaches the junction between the IVC and the RA. The reinfusion cannula (outflow) is inserted into the right internal jugular vein and then advanced through the superior vena cava and into the RA. 

The femoral-femoral VV-ECMO is also known as a double VV-ECMO cannulation. The drainage cannula is inserted into the femoral vein and advanced to the IVC. The reinfusion cannula is inserted into the contralateral femoral vein and advanced up to the RA. 

Dual lumen cannula VV-ECMO is a single cannula with a double lumen. It is inserted into the right internal jugular vein and advanced till the cannula tip reaches the IVC. The placement is more difficult than the previous two catheters and would require imaging (fluoroscopy) [4].

The final configuration type is femoral-femoral-jugular VAV-ECMO. It is also known as triple cannulation therapy and is utilized in patients for whom traditional VV-ECMO is not ideal for supportive therapy. An example could be a patient initially on VV-ECMO who develops cardiogenic shock, therefore, requiring venoarterial extracorporeal membrane oxygenation therapy [8]. The drainage cannula is inserted into the femoral vein and advanced till the cannula tip reaches the IVC. The reinfusion cannula is inserted into the internal jugular vein and is advanced till the cannula tip reaches the RA. In order to switch to VAV-ECMO, an additional cannula is inserted into the femoral artery and that is connected to the existing line using a Y shaped connector. 

Vascular access

Before inserting the cannulas for drainage and reinfusion, the vascular anatomy needs to be examined for underlying vascular disease or caval filter [4]. This can be done with an echocardiogram or ultrasound.

Prior to cannula placement, a bolus of heparin (approximately 50-100 units/kilogram) is given. There are four methods of cannula insertion. They are the cut-down exposure technique, the percutaneous Seldinger technique, the Semi-Seldinger technique, and by direct cannulation of the RA and aorta during a thoracotomy. Using real-time ultrasound guidance for cannulation has also become a standard of practice and is used during cannula insertion [9].

Cut-down Exposure Technique

The cut-down exposure technique is preferred for neonates and small children. This method is typically performed in the ICU or with an operating room (OR) team, as it requires fully sterile conditions. The patient is placed under sedation with muscle relaxation in order to prevent spontaneous breathing, which can lead to the formation of an air embolus. The skin is locally anesthetized and the tissues are separated to expose the vessels. The proximal targeted vessel is then occluded with a vascular clamp, opened, then is cannulated [4].

Percutaneous Seldinger Technique

The percutaneous technique involves using Seldinger technique for puncture of the needed vessel following its. In case the patient needs to be switched over to the cut-down exposure technique, it is important to perform the percutaneous cannulation in the ICU or the OR. The first step is to insert a small typical sized intravascular catheter (18-22 gauge). After the catheter is placed, a guidewire is passed through the catheter. The opening is then slowly enlarged with the help of serial dilators. Between dilators, the involved vessel should be occluded to prevent bleeding and wire should be checked to make sure it does not need to be replaced (bent wire must be removed). With the help of the guidewire, the cannula is gently inserted into the vessel. Ultrasound or fluoroscopic imaging can be used to help with insertion technique.

Semi-Seldinger Technique

The Semi-Seldinger technique involves cut-down exposure of the vessel combined with the Seldinger technique. The advantage of the Semi-Seldinger technique compared to the percutaneous Seldinger technique is that it has a faster insertion and a more flexible approach. The Semi-Seldinger technique for vascular access is performed in the ICU, OR or catheterization laboratory under anesthesia and after sterile preparation [4]. The cut-down technique exposes the targeted vessel without dissection. After exposure of the vessel, a small 20 gauge intravascular catheter is inserted into the vessel through the skin and distal to the incision. The placement of the catheter in the vein is confirmed by aspirating a small amount of venous blood. Once that is confirmed, the catheter is used to pass a guidewire through it and into the vein. Once the guidewire is inserted, serial dilators are used to enlarge the opening and eventually the cannula is placed using the guidewire for coordination. The wound edges are then closed over the cannula for stability.

Complications

Like any other central line insertion, VV-ECMO therapy can have complications. These include bleeding, deep venous thrombosis, possible stroke, infection, intravascular catheter breaking causing addition trauma, lost or broken guidewire, and others. With the increased use of ultrasound imaging during insertion, these complications have been greatly reduced.

## Conclusions

The material reviewed in this paper focuses on VV-ECMO therapy. It goes into detail regarding the principle, indication, contraindication, configurations, vascular access, and complications. It also addresses VAV-ECMO therapy, which is a new configuration of VV-ECMO therapy. Despite these components being addressed, larger and more prospective studies are needed to understand the true benefits of VV-ECMO therapy. This is a review article to allow busy, practicing physicians to have a cumulative view of our current situation regarding VV-ECMO therapy.
